# Ferroelectric hybrid organic–inorganic perovskites and their structural and functional diversity

**DOI:** 10.1093/nsr/nwac240

**Published:** 2022-11-02

**Authors:** Tie Zhang, Ke Xu, Jie Li, Lei He, Da-Wei Fu, Qiong Ye, Ren-Gen Xiong

**Affiliations:** Jiangsu Key Laboratory for Science and Applications of Molecular Ferroelectrics, Southeast University, Nanjing211189, China; Institute for Science and Applications of Molecular Ferroelectrics, Key Laboratory of the Ministry of Education for Advanced Catalysis Materials, Zhejiang Normal University, Jinhua321004, China; Jiangsu Key Laboratory for Science and Applications of Molecular Ferroelectrics, Southeast University, Nanjing211189, China; Jiangsu Key Laboratory for Science and Applications of Molecular Ferroelectrics, Southeast University, Nanjing211189, China; Jiangsu Key Laboratory for Science and Applications of Molecular Ferroelectrics, Southeast University, Nanjing211189, China; Institute for Science and Applications of Molecular Ferroelectrics, Key Laboratory of the Ministry of Education for Advanced Catalysis Materials, Zhejiang Normal University, Jinhua321004, China; Jiangsu Key Laboratory for Science and Applications of Molecular Ferroelectrics, Southeast University, Nanjing211189, China; Jiangsu Key Laboratory for Science and Applications of Molecular Ferroelectrics, Southeast University, Nanjing211189, China

**Keywords:** ferroelectrics, hybrid perovskite, molecular design, photoelectric effect, piezoelectricity

## Abstract

Molecular ferroelectrics have gradually aroused great interest in both fundamental scientific research and technological applications because of their easy processing, light weight and mechanical flexibility. Hybrid organic–inorganic perovskite ferroelectrics (HOIPFs), as a class of molecule-based ferroelectrics, have diverse functionalities owing to their unique structure and have become a hot spot in molecular ferroelectrics research. Therefore, they are extremely attractive in the field of ferroelectrics. However, there seems to be a lack of systematic review of their design, performance and potential applications. Herein, we review the recent development of HOIPFs from lead-based, lead-free and metal-free perovskites, and outline the versatility of these ferroelectrics, including piezoelectricity for mechanical energy-harvesting and optoelectronic properties for photovoltaics and light detection. Furthermore, a perspective view of the challenges and future directions of HOIPFs is also highlighted.

## Teaser

This review discusses the structural design and superior performance of organic–inorganic hybrid perovskite ferroelectrics and proposes their opportunities, challenges and future molecular ferroelectric development directions.

## INTRODUCTION

Ferroelectric materials, whose spontaneous polarization (*P*_s_) can be reversed or reoriented by an external electric field, have been developed for ∼100 years. Since Rochelle salt (potassium sodium tartrate tetrahydrate) [1] was discovered as the first ferroelectric, thousands of ferroelectrics have been discovered based on inorganic oxides [2], organic compounds [3], liquid crystals [4], polymers [5] and organic–inorganic hybrids [6]. Nevertheless, the rapid development of ferroelectrics in the past decades is mainly credited to the prosperity of ferroelectric inorganic oxide perovskites because of their relatively high stability and excellent ferroelectric properties. In recent years, molecular ferroelectrics (hybrid compounds and organic compounds) have reentered our vision due to their ease of solution processing, low cost, environmental friendliness, biocompatibility and homochirality (can expand the study of ferroelectric spintronics and polarized light that is difficult for inorganic ceramics [7,8]). Meanwhile, some significant breakthroughs in the most important figures (Curie temperature—*T*_C_, *P*_s_, piezoelectric coefficient*—d*_33_ and pyroelectric coefficient) of ferroelectrics have also been made in molecular ferroelectrics [8]. For example, organic ferroelectric croconic acid [9] and diisopropylammonium bromide (DIPAB) [10] have challenged the high *P*_s_ and *T*_C_ of inorganic ceramics, which has greatly promoted the development of molecular ferroelectrics. However, most organic molecular ferroelectrics suffer from weak piezoelectricity and instability, which are far lower than for inorganic ferroelectrics, hindering their progress.

Recently, as an important class of molecular ferroelectrics, hybrid organic–inorganic perovskite ferroelectrics (HOIPFs) with large piezoelectricity coefficient and *P*_s_, high *T*_C_ and multiple polar axes features are gradually appearing on the horizon, breaking the deadlock. Despite the relatively weak *P*_s_ or other figures of merit of ferroelectricity (fatigue, slower frequency) in HOIPFs compared to inorganic ferroelectrics, excellent examples of low-toxicity lead/metal-free HOIPFs are emerging [11]. Their good ferroelectricity, piezoelectricity and the advantage of a tunable chemical structure offer promise for the future of high-performance, low-cost fabrication, flexible, green and environmentally friendly ferroelectric devices [12]. Among them, the superior piezoelectricity of HOIPFs is particularly striking. It is well known that piezoelectricity usually arises in crystalline materials without inversion symmetry (except for the 432 point group) by the interconversion between electric voltage and mechanical stress [13]. But different from inorganic ferroelectrics, the main mechanism of piezoelectricity in HOIPFs is the electric dipole moment induced by the coexisting dipole transition of organic cations and octahedral distortion of inorganic skeletons under strain. Thus the piezoelectricity of HOIPFs is usually better than that of the other molecular ferroelectrics owing to the relatively large electric polarization displacement changes of HOIPFs [14]. Also, the ferroelectric polarization rotation and morphotropic phase boundary can enhance their piezoelectricity, similarly to those found in inorganic ferroelectrics [15–17]. Therefore, based on the above, regulating the structure is significant for obtaining the target performance ferroelectrics. Fortunately, HOIPFs with diverse tunable structures have infinite room as potential candidates for next-generation medical devices, micromechanical sensors or energy-storage devices.

Hybrid organic–inorganic perovskites (HOIPs) usually combine multiple advantages to create a material that ideally has greater functionality than the sum of its parts, like 1 + 1 > 2 [18]. Initially, HOIPs are limited to a typical ABX_3_ (A = organic anions, B = metal ions and X = halogens, CN^−^, N_3_^−^, N(CN)_2_^−^, NO_2_^−^, HCOO^−^ and BF_4_^−^) chemical formula structure. Then HOIPs expand to more structures, such as ABX_4_, A_3_B_2_X_9_ A_2_A′*_n_*_–__1_B*_n_*X_3_*_n_*_+1_, A_2_BB’X_4_ (double perovskite), ABX_5_ and ABX_6_, etc. [18–21]. How to get HOIPFs? It is essential first to understand the origin of ferroelectricity. The main origin of HOIPFs is the orientation or position changes of molecules/ions. And the most-encountered ferroelectric phase transitions mechanism includes an order–disorder or mixed order–disorder and displacive type [22–30]. Specifically, the origin of their ferroelectric behavior is not only attributed to the movement (canting or changes in ordered states) of light organic cations; the distortion and vibration of inorganic skeletons also play an important role [31]. The coordination bond, hydrogen bond, halogen–halogen effect and van der Waals effect form ‘softer’ lattices, leading them to have more active phononic states and an enhanced role for lattice vibrations. The organic part, inorganic part and their interaction could integrate and influence each other, resulting in the ferroelectric phase stability and the generation of polarity in the lattice. Lately, research on the contribution of collective atomic vibrations to ferroelectricity and the dynamically extended lattice effect has expanded the understanding of mechanisms of ferroelectricity [32]. The above undoubtedly provides a theoretical guide for us to design HOIPFs. Structurally, this also shows the importance of tuning the A-, B- and X-sites. In the past decades, modifying or doping the ionic primitives at the A-, B- and X- sites is the main chemical synthesis method in the design of hybrid ferroelectrics [12]. Benefitting from the contributions of many research groups and empirical mechanism of ferroelectrics, the semi-empirical chemical strategy involving a quasi-spherical strategy [33], momentum matching [34], homochirality [35], spatial confinement effect [36], H/F substitution [37] and a halogen regulation strategy [38] have developed in the process. As such, the amount of hybrid perovskite ferroelectrics with diverse structures was greatly increased and a wide variety of multifunctional HOIPFs was successfully synthesized. The detailed information for some typical ferroelectrics is shown in Table 1 and Supplementary Table S1. For example, lead-free solid solution ferroelectric (TMFM)*_x_*(TMCM)_1–_*_x_*CdCl_3_ (TMFM = trimethylfluoromethyl ammonium, TMCM = trimethylchloromethyl ammonium, 0 ≤ *x* ≤ 1) have excellent piezoelectric properties (high *d*_33_) due to the existence of the morphotropic phase boundary. At *x* = 0.26, the piezoelectric coefficient *d*_33_ can reach ∼1540 pC N^−1^ (pC N^−1^ = pico-coulombs per newton) comparable to the high-performance inorganic ceramic perovskites [16]. This is another great advance in the development of molecular ferroelectrics.

**Table 1.** Typical hybrid organic–inorganic perovskite ferroelectrics.

					
Compounds	*d*_33_ (pC N^−1^)	*P*_s_ (μC cm^−2^)	*T*_C_ (K)	Transformation	Ref
					
MAPbI_3_		7.2	330	*I*4*cm* to *Pmm*	[39]
MPSnBr_3_		4.5	314 357	*Pc* to *Pna*2_1_ to *Pm*3*m*	[40]
(MDABCO)RbI_3_		6.8	430	*R*3 to *P*432	[33]
[(CH_3_)_3_NOH]_2_[KFe(CN)_6_]		0.58	402	*Cc* to *Fmm*	[41]
(4,4-difluorocyclohexylammonium)_2_PbI_4_			377	*Cmc*2_1_ to *Pbca*	[42]
[*R(S)*-*N*-(1-phenylethyl)ethane-1,2-diaminium]_2_PbI_4_		0.15	389	*P*2_1_ to *P*2_1_2_1_2_1_	[43]
(2-fluorobenzylammonium)_2_PbCl_4_		1.52	448	*Cmc*2_1_ to *I*4*/mmm*	[44]
(BA)_2_(MA)_2_Sn_3_Br_10_	17.1	11.76	318	*Cmc*2_1_ to *Cmcm*	[45]
(AMP)(MA)Pb_2_I_7_		3.7		*Cc*	[46]
MDABCO–NH_4_I_3_	14	22.0	448	*R*3 to *P*432	[47]

MA, methylammonium; MP, methyl phosphonium; MDABCO, *N*-methyl-1,4-diazoniabicyclo(2.2.2)octane; [(CH_3_)_3_NOH]^+^, trimethylammonium-*N*-oxide; BA, n-butylammonium; AMP, 4-(aminomethyl)piperidinium; PXRD, powder X-ray diffraction; μC cm^−2^, microcoulombs per square centimeter.

By virtue of the development of the ferroelectric quantum theory and Berry phase calculation [48], their semiconducting properties are also focused like the parameters related to ferroelectricity. With the development of inorganic semiconducting ferroelectrics [49], semiconducting properties imparted by HOIPFs promote the development of their optoelectronic applications, providing a new platform for the next-generation soft functional ferroelectric materials. Some of them (Pb-based or lead-free) showed excellent semiconducting properties (easily tunable bandgap and energy structure, high carrier mobility/absorption coefficient and long carrier lifetime) comparable to or even surpassing those of inorganic semiconducting ferroelectrics such as BiFeO_3_ [49,50]. Moreover, distinguished luminescent properties (high-purity luminescence, wider luminescence range, high photoluminescence efficiency) [51] and circular polarization luminescent enantiomorphic have been reported by photoferroelectric (cyclohexylaminium)_2_PbBr_4_ [52], (*R*)- and (*S*)-3-(fluoropyrrolidinium)MnBr_3_ [53] and other functional ferroelectrics [54–56]. More than above, since the extensive exploration of the relationship between *P*_s_ and photoresponse effects of HOIPFs [57], multifunctional hybrid ferroelectric systems have been established [58] and broadened some new areas of ferroelectric optoelectronic application, such as bulk photovoltaic and self-powered photodetection [59]. Regrettably, their practical application is still being explored including ferroelectric information storage, piezoelectric energy storage, sensing and new-trend optoelectronic devices. The main question is that they lack high-quality crystals and usually exhibit mechanical weakness, low stability and short resistivity to environments. Notably, although their physical flexibility is not comparable to polymers, the preparation of films has fewer disadvantages compared to the complex, energy-consuming and time-consuming preparation of inorganic ferroelectric films. And they can be used without long pretreatments like polymers or some ceramics. This makes HOIPFs attractive in flexible devices. Moreover, hybrid perovskite ferroelectrics with chiral organic cations will promote the integration and development of ferroelectricity and chirality [7]. Overall, owing to their diverse and valuable properties and functions, HOIPFs should continue to be explored in fundamental research and practical applications.

**Scheme 1.**
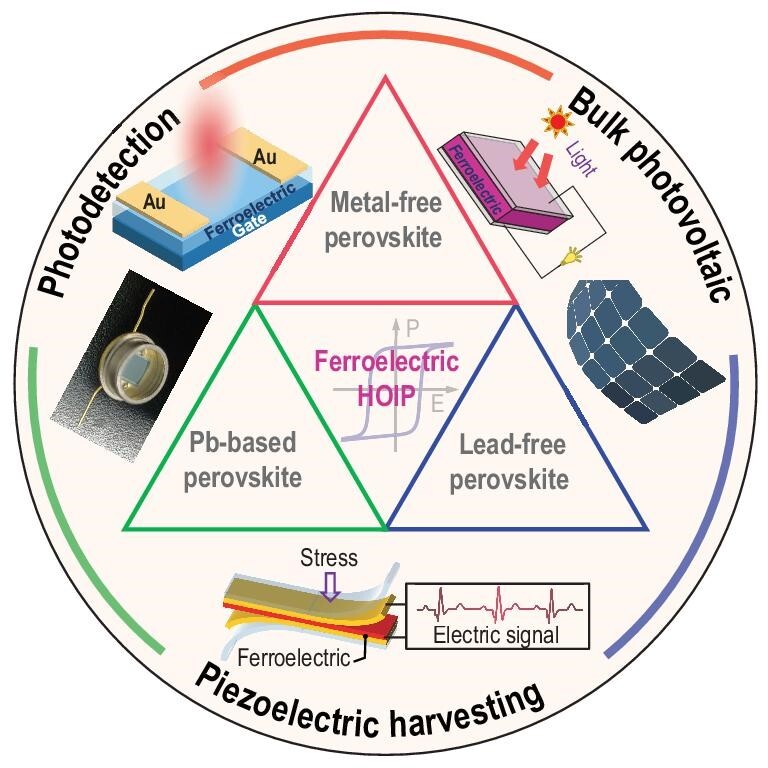
Schematic illustration of classification and functional application of hybrid organic–inorganic perovskite ferroelectrics.

Based on the above advantages and challenges in the field of HOIPFs [11,34,37,60,61], a systematic discussion and summary of their structure–property relationships are necessary for guiding their development. In this review, we seek to discuss the recent developments in the chemical design and performance optimization of HOIPFs, providing a theoretical platform to present the relationship between the structure and properties (ferroelectricity, piezoelectricity and photoresponse). Meanwhile, the challenges and advantages are discussed to guide the direction of overcoming restrictions for possible research directions in the future. In the following, this review involves three sections: (i) different B-site structures including Pb-based perovskite, lead-free perovskite and metal-free perovskite ferroelectrics; (ii) functional applications of HOIPFs, highlighting the importance of structure–property relationships; and (iii) outlook of hybrid ferroelectrics (Scheme 1). We envision that such hybrid materials will hold excellent performance in sensors, energy storage/conversion, photocatalysis and photovoltaics/detection.

## PB-BASED PEROVSKITE FERROELECTRICS

**Figure 1.**
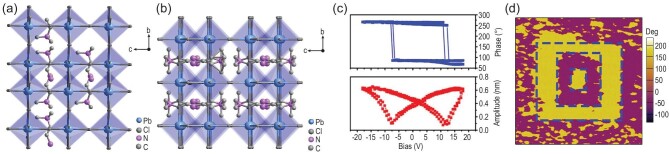
The packing crystal structure of 3D perovskite (TMAEA)Pb_2_Cl_6_ at (a) 293 K and (b) and 423 K. (c) PFM switching spectroscopy measured on an arbitrary point of the thin film. Adapted with permission from [66]. Copyright 2020, American Chemical Society. (d) Box-in-box domain pattern created by three-step electrical poling. Adapted with permission from [66]. Copyright 2020, American Chemical Society.

For hybrid perovskites, A-site represents MA, MP, [(CH_3_)_4_ N]^+^ (tetramethylammonium), [C(NH_2_)_3_]^+^ (guanidium), [(CH_3_)_3_NOH]^+^ (trimethylammonium-N-oxide) and so on; B-site is the common metal cation (Pb^2+^, Sn^2+^, K^+^, Zn^2+^, Rb^+^, Cr^3+^); and X-site is halogens (Cl^−^, Br^−^, I^−^) or polyatomic anions [CN^−^, N_3_^−^, HCOO^−^, BF_4_^−^ and N(CN)_2_^−^] [62]. Lead-based hybrid perovskites are one of the more widely studied branches. From zero-dimensional (0D) to 3D structures, they have diverse arrangements that endow them with diverse functions [63]. The star material MAPbI_3_ has been investigated to exhibit a polar crystal structure and ferroelectric domain shifting by Breternitz [64]. In 2017, Cahen *et al.* proved that MAPbI_3_ [65] is ferroelectric with a *T*_C_ of ∼330 K and changes from a ferroelectric phase (space group *I*4*cm*) to a paraelectric phase (space group *Pm*$\bar{3}$*m*). Recently, a 3D lead-based perovskite ferroelectric (TMAEA)Pb_2_Cl_6_ (TMAEA = 2-trimethylammonium ethyl ammonium) was reported [66], which consists of the corner- and edge-sharing PbCl_6_ octahedron combined 3D framework (Fig. 1a and b). The combination of ferroelectricity and promising semiconductor properties is reflected in (TMAEA)Pb_2_Cl_6_. (TMAEA)Pb_2_Cl_6_ exhibits a transition from the ferroelectric phase (space group *Pma*2) to the paraelectric phase (space group *Pmma*) at a *T*_C_ of 412 K. The hysteretic phase loops and typical butterfly shape of the amplitude loops evidence its robust ferroelectric polarization behavior (Fig. 1c). At the same time, piezoresponse force microscopy (PFM) measurements were used to examine the ferroelectricity in its thin-film samples. The following domain reversal can be achieved by changing the moving path and thus changing the direction of the trailing field. The written domain can be switched back and forth by moving the biased tip along the black dashed lines with voltages of +20 and +19 V. Finally, a box-in-box domain pattern can be generated (Fig. 1d), which further confirms the switchable polarization of (TMAEA)Pb_2_Cl_6_.

**Figure 2.**
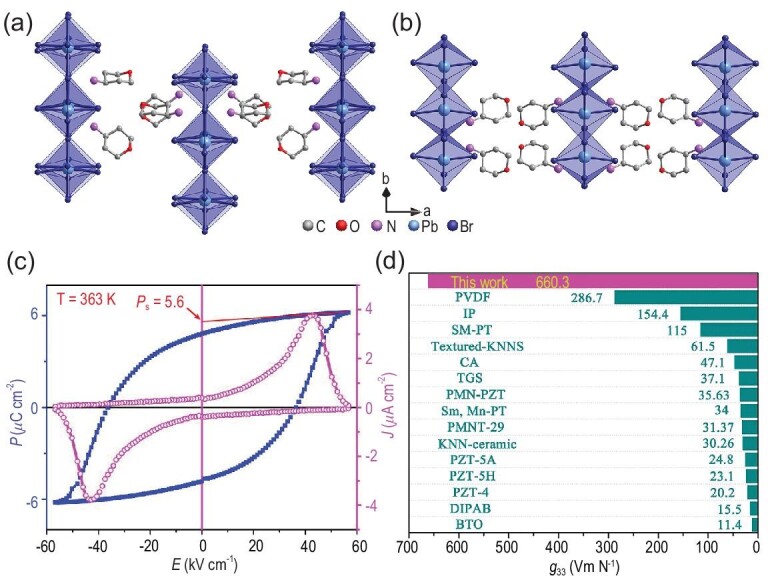
Packing view of the crystal structure of (ATHP)_2_PbBr_4_ layered perovskite at (a) 293 K and (b) 443 K. (c) *P*−*E* hysteresis loop of (ATHP)_2_PbBr_4_ was measured using the double-wave method at 300 K. Adapted with permission from [67]. Copyright 2020, American Chemical Society. (d) Comparison of piezoelectric voltage coefficients of (ATHP)_2_PbBr_4_ with some inorganic single crystals, textured ceramics, polymers and molecular ferroelectrics. Adapted with permission from [67]. Copyright 2020, American Chemical Society.

Then, two-dimensional (2D) hybrid ferroelectric (BA)_2_PbCl_4_ was reported by Luo *et al.* [51]. It shows a *P*_s_ value of 2.1 μC cm^−2^ and *T*_C_ of 328 K. Determination of the variable temperature single-crystal structure confirms a structural phase transition at *T*_C_ from the centrosymmetric space group *Cmca* in paraelectric phase to the polar space group *Cmc*2_1_ in ferroelectric phase. It is speculated that this symmetry breaking is related to the reorientation of dynamic organic cations. By adjusting the A-site moieties, more 2D hybrid perovskite ferroelectrics were studied. (ATHP)_2_PbBr_4_ (ATHP = 4-aminotetrahydropyran) was reported, exhibiting excellent performance [67]. From a structural perspective, the infinitely interlaced layers of PbBr_6_ octahedra are separated by ATHP cations (Fig. 2a and b). The ATHP cation is located almost in the center of the parallelogram diagonal, resulting in the staggered conformation of the inorganic lattice. At 293 K, (ATHP)_2_PbBr_4_ crystallizes in the space group *Cmc*2_1_. The polarization electric field (*P−E*) hysteresis loop is measured by utilizing the double-wave method at 363 K (Fig. 2c). (ATHP)_2_PbBr_4_ has predominant macroscopic ferroelectric polarization with a high *T*_C_ of 503 K beyond BaTiO_3_ (393 K) and an extremely large piezoelectric voltage coefficient (*g*_33_) of 660.3 × 10^−3^ V m N^−1^ that is much higher than that of high-end PZT [Pb(Zr, Ti)O_3_] (∼20−150 × 10^−3^ V m N^−1^) (Fig. 2d).

To avoid the randomness and blindness of searching for perovskite ferroelectrics using the conventional method, a design strategy of fluorine modification is employed. The H/F substitution where hydrogen is replaced by large electronegativity fluorine atoms causes the charge redistribution with a concomitant extendibility of the dipole moment [30]. And the hydrogen and fluorine atoms have a similar spatial parameter, which ensures no large structural changes. Moreover, organic cations may be tilted due to the distortion of the inorganic framework by the change of the organic parts causing the polar alignment of the lattice. The first fluorinated 2D lead–iodide perovskite ferroelectrics is (DFCHA)_2_PbI_4_ (DFCHA = 4,4-difluorocyclohexylammonium) using this approach [42]. The lead–iodide and the DFCHA cation are stacked alternatively to form a layered 2D perovskite structure. The symmetry of the material (DFCHA)_2_PbI_4_ shifts from a polar *Cmc*2_1_ to a nonpolar *Pbca* group at 377 K of *T*_C_. Half of the DFCHA cations are reoriented during the phase transition, breaking the inversion symmetry, so macroscopic polarization can be expected in this arrangement.

Besides, the introduction of chirality on organic cations is another effective way to design 2D HOIPFs. Zeng *et al.* introduce a methyl group and chiral site into the organic cation of the parent (*N*-benzylethane-1,2-diaminium)PbI_4_ and have successfully synthesized homochiral lead–iodide perovskite ferroelectrics [(*R*)-*N-*(1-phenylethyl)ethane-1,2-diaminium]PbI_4_ and [(*S*)-*N-*(1-phenylethyl)ethane-1,2-diaminium]PbI_4_ [43]. They both undergo paraelectric–ferroelectric transition at ∼389 K of *T*_C_. At 293 K, this pair of perovskites show a monoclinic chiral–polar *P*2_1_, but when the temperature rises to 423 K, they go to an orthorhombic *P*2_1_2_1_2_1_. Thus, the incorporation of homochiral organic molecules may contribute to the crystallization of the 2D HOIP into polar space groups.

In this section, we briefly describe the Pb-based HOIPFs. The A-site organic cation motion and inorganic skeleton distortion cooperatively influence their ferroelectric phase stability and the relationship between the dynamics of the extended lattice and local molecular groups [32]. Hence regulating the A-site and X-site is crucial for designing diverse Pb-based ferroelectrics with expected functionality and future applications. Meanwhile, due to the toxicity and instability of lead elements, it is necessary to improve the stability of Pb-based hybrid perovskite ferroelectrics and further develop lead-free perovskites.

## LEAD-FREE PEROVSKITE FERROELECTRICS

The challenges of the toxicity and instability of lead have led to the design of novel lead-free ferroelectric materials. Over the last few years, lead-free hybrid perovskite ferroelectrics have drawn increasing attention owing to their environmental friendliness and excellent ferroelectric properties. Their exceptional structural tunability also enables the formation of various types of crystal structures ranging from 3D to 0D. Among them, the B-site metals are mainly Mn, Cd, Cu, Zn, Ni, Cr, Sn, Bi and Sb [14,40,68–77].

Among the 0D lead-free HOIPFs, the reported metal cations include Sb and Bi. In 2016, Luo and co-workers systematically investigated the 0D perovskite ferroelectrics (*N*-methylpyrrolidinium)_3_Sb_2_Cl_9−9_*_x_*Br_9_*_x_* (*x* = 0−1). Taking the (*N*-methylpyrrolidinium)_3_Sb_2_Br_9_ as an example, it undergoes the phase transition from *R*3*c* (ferroelectric phase) to *R*$\bar{3}$*c* (paraelectric phase) at 322 K and presents a large ferroelectric *P*_s_ of ∼7.6 μC cm^−2^ [74]. The crystal structure contains a discrete inorganic unit (Sb_2_Br_9_)^3−^ and three organic cations. In this compound, the lone-pair electrons of Sb^3+^ sterically interfere with the motion of Sb_2_X_9_^3−^ octahedra, resulting in the Sb atom being away from the octahedral center to induce ferroelectricity. Besides, the perovskite ferroelectric (FA)_5_Sb_2_Br_11_ (FA = formamidinium) was discovered by K. Mencel and A. Piecha-Bisiorek in 2020 [78]. It crystallized in space group *P*2_1_ and has a ferroelectric phase transition at 163 K. The *P*_s_ and coercive field (*E*_c_) values reach 3.0 μC cm^−2^ and 370 kV cm^−1^, respectively. Its *P*_s_ originates from the molecular ordering of the polar organic cation and the total polar distortion of the bi-octahedral unit.

**Figure 3.**
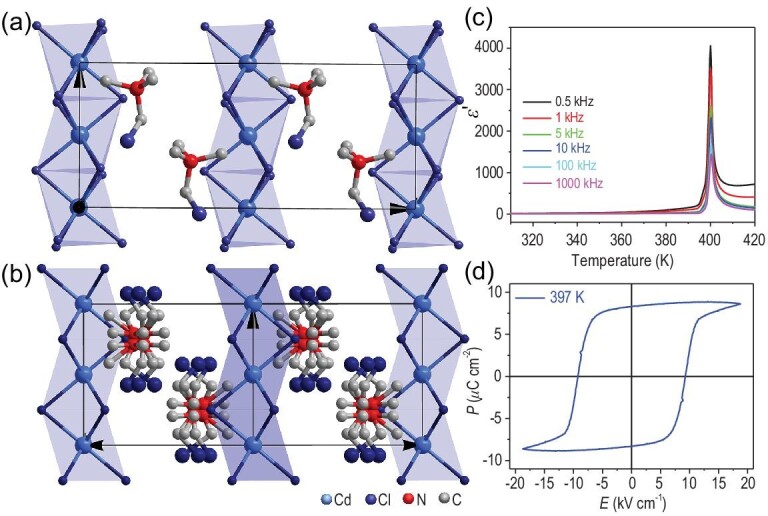
Crystal structures of TMCMCdCl_3_ in (a) the ferroelectric phase and (b) the paraelectric phase. (c) Temperature dependence of the dielectric permittivity. Adapted with permission from [15]. Copyright 2017, American Association for the Advancement of Science. (d) *P*−*E* hysteresis loop measured from a Sawyer−Tower circuit. Adapted with permission from [15]. Copyright 2017, American Association for the Advancement of Science.

As for the one-dimensional (1D) lead-free HOIPFs, they adopt a hexagonal BaNiO_3_-like structure containing infinite face-sharing chains of metal–halide octahedra and A-site discrete cations between the chains. It was reported that the 1D hybrid perovskite ferroelectric [(CH_3_)_4_ N]CdCl_3_ crystallizes in the centrosymmetric space groups *P*6_3_/*m* and *P*6_3_/*mmc* at room temperature and 410 K, respectively [79]. Hence, the [(CH_3_)_4_ N]CdCl_3_ does not crystallize in the polar point group and is not ferroelectric. In 2019, the first 1D HOIP phosphonium-based ferroelectric [(CH_3_)_4_P]CdCl_3_ was discovered by replacing the N atom of [(CH_3_)_4_ N]^+^ with a heavier P atom [80]. It has a relatively high *T*_C_ of 348 K and remnant polarization (*P*_r_) of 0.43 μC cm^−2^ compared to its isostructural compound [(CH_3_)_4_ N]CdBr_3_. The symmetry shifts from a chiral *P*6_3_ to a centrosymmetric *P*6_3_/*m* group at *T*_C_. Single-crystal structural analysis and solid-state nuclear magnetic resonance studies reveal that the ferroelectric phase transition originates from an order–disorder transition of the [(CH_3_)_4_P]^+^ cations. Besides, a Cd-based hybrid perovskite ferroelectric TMCMCdCl_3_ was discovered by substituting one methyl of the [(CH_3_)_4_ N]^+^ cation with chloromethyl [15] (Fig. 3). Its ferroelectric phase belongs to the polar space group *Cc* and the paraelectric phase is centrosymmetric space group *P*6_3_/*mmc*. The phase transition with the Aizu notion of 6/*mmmFm* is triggered by dynamic changes in the organic cations from a fully disordered paraelectric phase to an ordered ferroelectric phase. Moreover, it exhibits a high *T*_C_ of 400 K and a large *P*_s_ of 6.0 μC cm^−2^. Recently, by replacing the B-site metal Cd atom with a divalent Cr atom, a Cr-based HOIP TMCMCrCl_3_ was found to show the coexistence of ferroelectricity and anti-ferromagnetism [54]. The variable temperature single-crystal structure confirms a paraelectric–ferroelectric phase transition at 397 K from the centrosymmetric space group *P*6_3_/*m* to the monoclinic polar space group *Pc*. The measured *P*_s_ and *P*_r_ are almost equal, at ∼3.6 μC cm^−2^. The ferroelectricity originates from the orientational ordering of TMCM cations and the magnetism is from the (CrCl_3_)^−^ framework.

**Figure 4.**
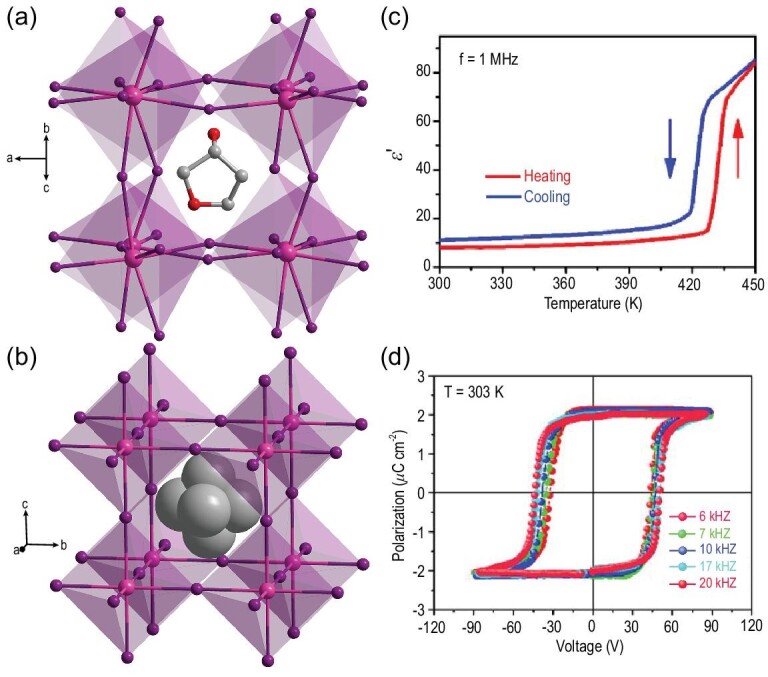
Crystal structures of (3-AP)RbBr_3_ in (a) the ferroelectric phase and (b) the paraelectric phase. (c) Temperature dependence of the dielectric permittivity. Adapted with permission from [81]. Copyright 2017, American Chemical Society. (d) *P*−*E* hysteresis loop measured from a Sawyer−Tower circuit. Adapted with permission from [81]. Copyright 2017, American Chemical Society.

3D lead-free perovskite ferroelectrics have also been discovered in Sn- and Rb-based hybrids. The first methylphosphonium-based MPSnBr_3_ exhibits a ferroelectric phase transition from polar *Pna*2_1_ to nonpolar *Pm*$\bar{3}$*m* group at a *T*_C_ of 357 K [40]. Similar to the MA, MP is a small cation, making it an excellent choice for the A-site to construct 3D ABX_3_ HOIP. Interestingly, MPSnBr_3_ is a multi-axial molecular ferroelectric with 12 polar axes, which is more than those of the classical inorganic perovskite ferroelectric BiFeO_3_ (4 polar axes) and BaTiO_3_ (3 polar axes). Both the P element and the N element with similar bond properties are listed in the VA group of the periodic table. The P element is in the third period and the MP has a larger volume and heavier mass than the MA. Thus the MP not only meets the need to build 3D stannous bromide perovskite ferroelectrics but also increases the rotational energy barrier to raise the phase-transition temperature. Moreover, the alkali-metal Rb^+^ was also a good candidate for the B in the ABX_3_ structure. A 3D perovskite ferroelectric 3-APRbBr_3_ (3-AP = 3-ammoniopyrrolidinium) was reported (Fig. 4) [81]. 3-APRbBr_3_ undergoes a distinct transition from the paraelectric space group *Pm*$\bar{3}$*m* to the ferroelectric space group *Ia*, and it possesses 12 equivalent ferroelectric axes. It has a high *T*_C_ of 440 K beyond that of BaTiO_3_. Similarly, in the larger RbI_3_ framework, *N*-methyl-1,4-diazoniabicyclo(2.2.2)octane (MDABCO) was selected to become the A-site cation, forming a new 3D HOIP ferroelectric (MDABCO)RbI_3_ [33]. This ferroelectric crystallizes in polar space group *R*3 and undergoes a ferroelectric phase transition at ∼430 K. Based on previous experience, the reorientation and ordering of organic cations may provide ferroelectric phase transitions [12].

Besides, a series of 2D lead-free HOIP halide ferroelectrics was constructed by replacing the B-site monometal with heterometal. Sun and co-workers reported the first 2D halide double perovskite ferroelectric (chloropropylammonium)_4_AgBiBr_8_ [82]. It exhibits ferroelectricity with *T*_C_ = 305 K and *P*_s_ = 3.2 μC cm^−2^. (chloropropylammonium)_4_AgBiBr_8_ belongs to the monoclinic system with a polar space group *Pc* at 270 K. The basic structure is a 2D monolayer perovskite skeleton consisting of AgBr_6_/BiBr_6_ octahedra with shared angles of heterogeneous metals. The AgBr_6_ octahedra have a spatially distorted configuration and the BiBr_6_ adopts an almost ortho-octahedral geometry. Meanwhile, the organic cations are located between two inorganic layers and the NH_4_^+^ moiety is connected to the perovskite by strong N*−*H···Br hydrogen bonds. At 343 K, the structure transforms to a centrosymmetric space group *Pbam* of the orthorhombic system and cations are partially disordered.

In this section, we briefly summarize lead-free HOIP ferroelectrics from different dimensions. They exhibit promising ferroelectric properties including high *T*_C_ and large *P*_s_. These finds signify an important step toward designing and exploring new lead-free HOIP ferroelectric materials.

## METAL-FREE PEROVSKITE FERROELECTRICS

Recently, metal-free organic perovskite ferroelectrics have emerged as a surprise in the perovskite ferroelectric family. Compared with traditional perovskites, metal-free perovskites not only adopt the ABX_3_ perovskite structure but take advantage of the tunability, diversity and mechanical flexibility of organic materials [19,83]. (i) Metal-free perovskites can effectively avoid potential metal toxicity due to the absence of metal elements; (ii) the organic part gives the structure greater flexibility than metal bonding; (iii) the unique chirality of molecular materials can introduce optical activity and give materials special optical rotation applications; (iv) when all metal cations in the material are replaced by organics, its characteristics will be more suitable for the current requirements of materials in the preparation of thin films, flexibility and other aspects [84].

**Figure 5.**
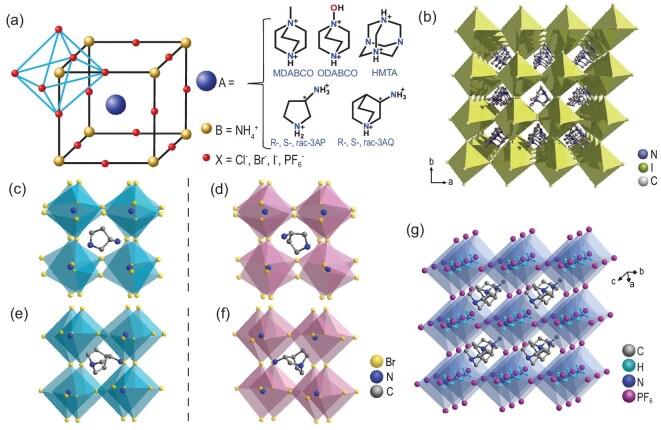
Chemical structure model of the metal-free perovskite ferroelectrics (a) A(NH_4_)X_3_. (b) The packing diagram of MDABCO–NH_4_I_3_ in the ferroelectric phase (adapted with permission from [47]. Copyright 2018, American Association for the Advancement of Science). The crystal structures of (c) *S*-3-AP–NH_4_Br_3_ and (d) its enantiomer *R*-3-AP–NH_4_Br_3_, (e) *S*-3-AQ–NH_4_Br_3_ and (f) its enantiomer *R*-3-AQ–NH_4_Br_3_ in the ferroelectric phases. (g) The packing structure of MDABCO–NH_4_(PF_6_)_3_ in the ferroelectric phase.

They have the general formula of A(NH_4_)X_3_, which is similar to 3D perovskite ferroelectrics. Structurally, different from the other perovskites, in metal-free perovskites, the A-site is a divalent quasi-spherical cation [A = 3-AP, *N*-*N*'-diazabicyclo(2.2.2)octonium (DABCO), hexamethylenetetramium (HMTA) and its derivatives, etc.], the B-site is the organic monovalent NH_4_^+^ and X is the halogen (Cl^−^, Br^−^, I^−^) or other diatomic anions (BF_4_^−^ or PF_6_^−^) [85,86]. As shown in Fig. 5a, the metal-free perovskite structure consists of a 3D network of corner-sharing (NH_4_)X_6_ octahedra with the divalent organic cations confined in the cages enclosed by the octahedral [47,87]. And the (NH_4_)X_6_ octahedra are mainly firmly connected by hydrogen bonds. The first metal-free perovskite (piperazinium)(NH_4_Cl_3_)·H_2_O was synthesized with the 3D corner-sharing [(NH_4_)Cl_6_] network [88]. Strictly speaking, it belongs to the A(NH_4_)X_3_C-type perovskite rather than the A(NH_4_)X_3_-type perovskite structure. And unfortunately, there were no observations of ferroelectricity.

Metal-free perovskite ferroelectrics were successfully synthesized by introducing the ‘quasi-spherical theory’ and symmetry-breaking principle through the A-site substitution strategy [34]. The hydrogen-bonding interaction between the A^2+^ cation and the negatively charged (NH_4_)X_3_ framework plays a huge role in the order–disorder phase transition, which is also unmatched by inorganic perovskites. In 2018, the Xiong group first reported a series of metal-free perovskite ferroelectrics using different A-site cations such as MDABCO, ODABCO [*N*-hydroxy-N′-diazabicyclo(2.2.2)octonium] and chiral molecule *R*/*S*-3-AP (*R*/*S*-3-ammoniopyrrolidinium) and 3-AQ (3-ammonioquinuclidinium) as shown in Fig. 5a [47].

Among the reported A(NH_4_)X_3_ perovskites, MDABCO–NH_4_I_3_ has a high Curie temperature of 448 K and a large *P*_s_ of 22 μC cm^−2^, which are comparable to those of the inorganic ferroelectric BaTiO_3_. At room temperature, it crystallizes in trigonal polar space group *R*3. In the cage-like framework, the quasi-spherical MDABCO cation is located in the cavities composed of distorted (NH_4_)I_6_ octahedrons. (Fig. 5b) The quasi-spherical geometry makes molecular reorientation easy, which is necessary for ferroelectric polarization reversal. As the temperature rises to 448 K (paraelectric phase), it transforms to the cubic nonpolar space group *P*432. The MDABCO cations undergo violent disorder rotation and the (NH_4_)I_6_ octahedrons become regular.

Moreover, based on the A-site substitution, some chiral metal-free perovskite ferroelectrics were successfully synthesized. In the 3D metal-free chiral perovskites (Fig. 5c–f), chiral 3-ammoniopyrrolidinium (3AP, Fig. 5c and d) and 3-ammonioquinuclidinium (3AQ, Fig. 5e and f) were employed to introduce chirality and the resulting *R*(or *S*)-3AP–NH_4_X_3_ and *R*(or *S*)-3AQ–NH_4_X_3_ belong to *P*2_1_ polar space group at room temperature (X = Cl^−^, Br^−^), thus enabling pyroelectric and ferroelectric properties. Therefore, compared with inorganic perovskites, the cations at the A-site also have a wide range of chemical diversity (especially polar or chiral cations) and structural flexibility, which enable metal-free perovskite ferroelectrics to achieve a variety of additional functions. Generally, homochiral molecules are crystallized in the five enantiomorphic polar point groups (*C*_1_, *C*_2_, *C*_3_, *C*_4_ and *C*_6_) and can form enantiomorphic crystals. The 3D metal-free chiral perovskite ferroelectrics can be regarded as an ideal target for developing new multi-axial ferroelectrics, which hold great potential for use in thin-film devices because of their minimum requirements for crystallinity and specific substrates. And optically active chiral compounds have potential applications in pharmaceuticals [89], the bio-industry [90], electro-optics and ferroelectric optics [35,84] and other fields [84].

Another method is the X-site substitution strategy. The X-site I^−^ in MDABCO–NH_4_I_3_ could be replaced by another diatomic anion such as PF_6_^−^. The Loh group reported a new metal-free ferroelectric perovskite MDABCO–NH_4_(PF_6_)_3_ (MNP3) [86], which has a coercive field (110 kV cm^−1^) that is larger than that of MDABCO–NH_4_I_3_ (12 kV cm^−1^) owing to its stronger intermolecular hydrogen bonding. Similar to the MDABCO–NH_4_I_3_ structure, the quasi-spherical MDABCO cation was located in the cavities composed of distorted (NH_4_)(PF_6_) octahedrons in the cage-like unit cell as shown in Fig. 5g. Based on the isotope experiments, the ferroelectric-to-paraelectric phase-transition temperature and coercive field are verified to be strongly influenced by hydrogen bonds. MDABCO–NH_4_(PF_6_)_3_ and DMDABCO–NH_4_(PF_6_)_3_ (D-MNP3 = deuterated-MNP3) both undergo a ferroelectric space group (*R*3) to the paraelectric space group (cubic *P*432) phase transition, which is the same as the reported metal-free perovskite ferroelectric MDABCO–NH_4_I_3_. The phase-transition temperatures of D-MNP3 and MNP3 are 320 and 301 K, respectively, which are lower than those of MDABCO–NH_4_I_3_ (448 K). Therefore, the X-site substitution strategy is an important method to enrich the composition and properties of the metal-free perovskite ferroelectrics family.

Taking MDABCO–NH_4_I_3_ as an example, metal-free perovskite ferroelectrics present other interesting properties and applications. Although the piezoelectric coefficient *d*_33_ [along the (1 1 1) direction] of metal-free ferroelectric MDABCO–NH_4_I_3_ is ∼14 pC N^−1^, the large piezoelectric strain components were observed by calculations. Wang's group studied the origin of polarization, electronic density of state, piezoelectric response and elastic properties of MDABCO–NH_4_X_3_ (X = Cl^−^, Br^−^, I^−^) by first-principles calculations [91]. By calculation, the *d*_x5_ values are 119, 248 and 178 pC N^−1^ for MDABCO–NH_4_X_3_ (X = Cl^−^, Br^−^ and I^−^), respectively. Meanwhile, it was found that the large value of *d*_x5_ is closely related to the large value of elastic compliance tensor *s*_44_. These results show that ferroelectrics MDABCO–NH_4_X_3_ have a large piezoelectric response with soft elastic properties. The large spontaneous polarization of MDABCO–NH_4_I_3_ mainly results from MDABCO and NH_4_^+^ being off-center relative to I ions.

In addition, extraordinarily large electrocaloric (EC) and elastocaloric (eC) strengths for (MDABCO)(NH_4_)I_3_ crystals and thin films, respectively, were found using experiments and calculations [92,93]. Chen's group predicted the ferroelectric (MDABCO)(NH_4_)I_3_ crystal, which has an extraordinarily large EC strength: isothermal Δ*S*_EC_/Δ*E* (electrocaloric strengths) and adiabatic Δ*T*_EC_/Δ*E* (electrocaloric strengths) for MDABCO are 18 J m kg^−1^ K^−1^ MV^−1^ and 8.06 K m MV^−1^, respectively, which are more than three times the largest reported values in BaTiO_3_ single crystals [94]. Hong's group predicted extraordinarily large eC strengths for the (111)-oriented metal-free perovskite ferroelectric (MDABCO)(NH_4_)I_3_ thin films by a thermodynamic phenomenological model. The predicted room temperature isothermal eC ΔS_eC_/Δσ (eC entropy change under unit stress change) and adiabatic eC ΔT_eC_/Δσ (eC temperature change under unit stress change) values for (MDABCO)(NH_4_)I_3_ are 60.0 J K^−1^ kg^−1^ GPa^−1^ and 17.9 K GPa^−1^, respectively, which are 20 times higher than those of ferroelectric oxides such as BaTiO_3_ thin films [92]. So the metal-free perovskite ferroelectrics were not only great electrocaloric materials but also excellent elastocaloric materials. The availability of high electrocaloric and large elastocaloric strength in materials is the key to achieving refrigeration in industrial applications. Metal-free perovskite ferroelectrics contribute to the development of new cooling technologies, reducing environmental damage from large quantities of refrigerants and improving energy efficiency. Therefore, metal-free ferroelectrics could be the best candidates for electrocaloric and elastocaloric applications.

Metal-free perovskite ferroelectrics also embrace special mechanical properties. The Kieslich group probed the mechanical properties including the bulk modulus, Young's modulus and hardness of (MDABCO)(NH_4_)I_3_ by combining high-pressure single-crystal X-ray diffraction and nano-indentation. With a similar bulk modulus, the Young's modulus of (MDABCO)(NH_4_)I_3_ (*E*_[111]_ = 14.7 GPa) is comparable to those of other Pb-based HOIPFs, such as MAPbI_3_ (*E*_[100]_ = 10.4 GPa), MAPbCl_3_ (*E*_[100]_ = 19.8 GPa) and FAPbI_3_ (*E*_[100]_ = 11.8 GPa) [95,96]. The Young's modulus was increased by the number of hydrogen-bonding interactions between organic cations and negatively charged 3D networks in the metal-free ferroelectrics. Interestingly, the great potential of (MDABCO)(NH_4_)I_3_ is similar to the situation in which the star MAPbI_3_ material was first discovered. And it is expected to promote further research into the metal-free perovskite ferroelectric family with the progress of MAPbI_3_ flexible photovoltaic devices.

Liu's group reported that metal-free ferroelectrics could be an excellent candidate for a biocompatible X-ray detector [97]. Unlike conventional 3D metal-based perovskites, the metal-free ferroelectric (MDABCO)(NH_4_)I_3_ crystal can easily get from an aqueous solution at room temperature and achieve both a 1.03-μs-long carrier lifetime and the formation of self-trapped excited states for luminescence, which contribute to a high photoluminescence quantum yield (PLQY, 53%) at room temperature and an X-ray sensitivity of ≤1997 ± 80 μC Gy cm^−2^ at 50 V of bias (highest among all metal-free detectors). These attributes make metal-free ferroelectrics an alternative to metal-based perovskites for biocompatibility and transient optoelectronics, and are more beneficial for their applications in wearable and lightweight devices.

In this section, we discuss the structure of A(NH_4_)X_3_-type metal-free perovskite ferroelectrics. They are all the classical ABX_3_ types. By A-site and X-site substitution strategy, a series of metal-free perovskite ferroelectrics could be obtained. And some metal-free perovskites show a high phase-transition temperature, large *P*_s_, large electrocaloric and elastocaloric strengths, special mechanical properties and excellent biocompatible X-ray properties. Therefore, metal-free perovskite ferroelectrics will be used in flexible devices, soft robotics and other applications that benefit from these extraordinarily excellent properties.

## FUNCTIONAL APPLICATION OF HOIPFs

Along with the development of diverse hybrid ferroelectrics, their functional application features such as piezoelectricity and semiconducting are also developing and attractive.

### Piezoelectricity in HOIPFs

Ferroelectrics is a subset of piezoelectric materials and piezoelectricity is one of the important macroscopic properties of ferroelectrics. HOIPFs have superior piezoelectric performance compared with other molecular ferroelectrics or even some inorganic piezoelectric ceramics. Therefore, it is necessary to discuss and summarize their excellent piezoelectric performance.

In terms of piezoelectrics, early interest is in inorganic ceramic perovskite ferroelectrics with robust properties such as PZT and BaTiO_3_, which are essential for high-voltage sources, sensors, actuators, frequency standards, vibration reducers and other applications [13,98]. Lately, with the demand for environmentally friendly, soft and flexible materials, HOIPFs with excellent piezoelectric performance (high *d*_33_, enhanced *T*_C_ and *P*_s_ or multi-polar axes) has attracted tremendous attention, especially non-lead HOIPFs [11]. Ferroelectricity can be obtained by replacing the A-site organic cations in designing hybrid perovskite ferroelectrics and their superior piezoelectric performance can also be improved by structural modification. In the last 10 years, a series of hybrid perovskite ferroelectrics with high-performance piezoelectrics was designed, approaching or even exceeding some inorganic piezoelectric ceramics (Table 2). For instance, lead-free perovskite ferroelectric TMCM–MnCl_3_ and TMCM–CdCl_3_ exhibit an excellent piezoelectric response (*d*_33_ = 185 pC N^−1^ and 220 pC N^−1^) which are close and even beyond that of traditional inorganic ceramics BaTiO_3_ (*d*_33_ = 190 pC N^−1^) [15]. More appealingly, the molecular perovskite ferroelectric solid solution (TMFM)*_x_*(TMCM)_1–_*_x_*CdCl_3_ has higher piezoelectricity (*d*_33_∼1540 pC N^−1^) that is larger than that of lead zirconate titanate [16], which greatly promotes the potential applications of the hybrid perovskite ferroelectrics in wearable piezoelectric devices. Another example, (RM3HQ)_2_RbLa(NO_3_)_6_ (RM3HQ = *R*-*N*-methyl-3-hydroxylquinuclidinium) also has a large piezoelectric response (106 pC N^−1^) for piezoelectric devices [99]. Particularly in 2018, the rise of organic perovskite ferroelectrics MDABCO–NH_4_I_3_, etc. [47] with excellent piezoelectricity brought an intriguing new area of organic ferroelectrics. These promising hybrid perovskite ferroelectric materials are expected to be applied in flexible piezoelectric energy-harvesting and other piezoelectric devices [45,71,100–103].

**Table 2.** Piezoelectric coefficient, *P*_s_ and *T*_C_ of various HOIPFs and selected inorganic oxide ferroelectrics.

				
Compounds	*d*_33_ (pC N^−1^)	*P*_s_ (μC cm^−2^)	*T*_C_ (K)	Refs.
				
PTMACdBr_3_*_x_*Cl_3(1−_*_x_*_)_	1000–4380 pm V^−1^(*d*_35_)	–	–	[14]
(TMCM)MnCl_3_	185	4.0	406	[15]
(TMCM)CdCl_3_	220–240	6.0	400	[15]
(TMFM)*_x_*(TMCM)_1–_*_x_*CdCl_3_ (*x* = 0.26)	1540	–	366.8	[16]
(BA)_2_(MA)_2_Sn_3_Br_10_	17.1	11.76	318	[45]
MDABCO–NH_4_I_3_	14	22	448	[47]
(ATHP)_2_PbBr_4_	76	5.6	503	[67]
(Me_3_NCH_2_CH_2_OH)CdCl_3_	3	17.1	393	[71]
MDABCO–NH_4_Cl_3_	119 (calculated *d*_x5_)	–	–	[95]
MDABCO–NH_4_Br_3_	248 (calculated *d*_x5_)	–	390	[95]
MDABCO–NH_4_I_3_	178 (calculated *d*_x5_)	–	446	[95]
(RM3HQ)_2_RbLa(NO_3_)_6_	106	0.28	285	[99]
(RM3HQ)_2_–NH_4_La(NO_3_)_6_	81	–	314	[99]
(TMCM)CdBr_3_	139	3.5	346	[101]
TMBM–MnBr_3_	112	3.5	433	[102]
DMAACdCl_3_	41	1.9	339	[103]
Selected conventional ferroelectrics				
LiTaO_3_	8	50	938	[104]
LiNbO_3_	6–16	71	1483	[105]
PbTiO_3_	47	50	763	[106]
BaTiO_3_	190	26	393	[107]
BiFeO_3_	70 pm V^−1^	90–100	1103	[108]
PZT	<750	–	–	[109]
PMN–PZT	1530	30	489	[110]
PMN–0.33PT	2400	–	425	[111]
Sm–PMN–PT	3400–4100	–	388	[112]

PTMA, *N*,***N***,*N*-trimethylanilinium; TMBM, trimethyl-bromidemethyl ammonium; [(CH_3_)_3_NCH_2_CH_2_OH]^+^, (2-hydroxyethyl)trimethylammonium; DMAA, *N,N*-dimethylallylammonium; PZT, Pb(Zr, Ti)O_3_; PMN–PT, Pb(Mg_1/3_Nb_2/3_)O_3_–PbTiO_3_; Sm–PMN–PT, Sm-doped Pb(Mg_1/3_Nb_2/3_)O_3_–PbTiO_3_. Table 3 shows the captions of some abbreviations and quantities in this article.

### Semiconducting properties of HOIPFs

**Figure 6.**
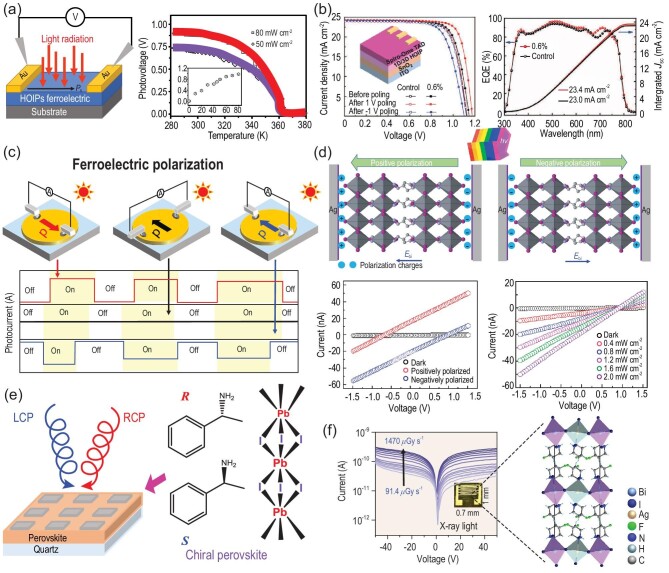
(a) Schematic for symmetrical top electrodes and temperature dependence of photovoltage. Inset: *y*-axis [voltage (V)] vs. *x*-axis [power (mW cm^−2^)]. Adapted with permission from [52]. Copyright 2016, Wiley-VCH. (b) Current density−voltage (*J–V*) curves of control and 1D/3D mixed perovskite solar cells (PSCs) before and after poling and external quantum efficiency (EQE) of control and 1D/3D mixed PSCs. (DFCHA)_2_PbI_4_ ferroelectric photovoltaic test. Adapted with permission from [120]. Copyright 2021, Wiley-VCH. (c) Measure the short-circuit photocurrent with electrodes parallel and perpendicular to the direction of ferroelectric polarization. Adapted with permission from [42]. Copyright 2019, Wiley-VCH. (d) Schematic of tunable photocurrent directions in the positive and negative polarization states for photoferroelectric. Current−voltage (*I−V*) characteristics with the inversion of polarization directions and light-intensity-dependent self-powered photoresponse properties of a negatively polarized (AMP)(MA)Pb_2_I_7_ (AMP = 4-(aminomethyl-piperidinium)device (−5 V for 5 min). Adapted with permission from [46]. Copyright 2020, American Chemical Society. (e) Scheme of the circularly polarized photodetector. Circularly polarized photodetectors distinguish left-handed and right-handed circularly polarized (LCP and RCP, respectively) light. Adapted with permission from [123]. Copyright 2019, Springer Nature. (f) *I–V* curves measured on (4,4-difluoropiperidinium)_2_AgBiI_8_ crystal detector at a 91.4- to 1470-μGy_air_ s^−1^ dose rate. Inset: the device of planar X-ray detector of the crystal. Adapted with permission from [124]. Copyright 2021, Wiley-VCH.

Optoelectronic materials, based on absorbing light to produce electrical signals, play an important role in optoelectronic devices. In recent years, HOIPs represented by the potential ferroelectric MAPbI_3_ have shown intriguing photovoltaic performance in solar cells and light detection, attributed to their potential polarization, domain structures and semiconducting properties [39], thus arousing much attention in the search for HOIPFs. On the one hand, they possess long carrier-diffusion lengths/spin lifetimes, high absorption coefficients, tunable band gaps and low defect density [18,113]. And photoexcited electron–hole pairs in HOIPFs can be transported and separated by their built-in electric field. As a result, it may make breakthroughs in low-power-consumption and high-performance photovoltaic and photodetection, especially in terms of high response speed, high conversion efficiency and high contrast [114–116]. On the other hand, they could have a wide structural tunability to construct a simple device without the p–n junction structure due to the polar structure-induced internal electric field, realizing low cost and miniaturization [117]. This is unmatched by other inorganic or polymer ferroelectrics. To date, several Pb-, Sn- and Bi-based ferroelectric semiconductors with excellent photoelectric performance have been reported [59], which are expected for efficient bulk photovoltaics and photodetection. For example, some 2D perovskite ferroelectrics [52,61,118] with a bulk photovoltaic effect have been successfully synthesized (Fig. 6a). Pb-based ferroelectric materials are mainly used because they possess a low trap density for the photoinduced charge carriers. Hybrid perovskite photo-ferroelectrics, such as layered bis(cyclohexylaminium)PbBr_4_ and (4,4-difluorocyclohexylammonium)_2_PbI_4_, exhibit an exceptional anisotropy of bulk photovoltaic effects [42]. HOIPFs assist in improving photovoltaic devices with a multi-layer structure [119,120] (Fig. 6b and c). Compared with their photovoltaics effect, research on hybrid perovskite ferroelectric photodetection has also made greater progress, which makes up the relatively small zero-bias photocurrent of traditional inorganic oxides in self-powered ferroelectric photodetectors. Among them, the discovery of 2D HOIPFs that can be applied to self-powered photodetectors is a milestone in the development of ferroelectric semiconductors. The integration of organic and inorganic components gives them a diverse structure, resulting in excellent charge transport and light absorption over a wide range from visible-blind ultraviolet and visible light to near-infrared light [46] (Fig. 6d). To remove toxic Pb metal, Bi-based and other low toxic perovskite ferroelectrics have been designed and investigated on their photodetector performance [121]. Unfortunately, the photoresponse is unsatisfactory compared with Pb-based materials. Moreover, the presence of chiral organics in HOIPFs can be used in circularly polarized light (CPL) detection [122], e.g. (*R*- and *S*-α-PEA)PbI_3_ (α-PEA: α-phenylethylamine) [123] (Fig. 6e). Although their ferroelectric properties have not been confirmed, there is an urgent need to explore the self-powered CPL detection of hybrid perovskite ferroelectrics. In addition, in terms of high-energy electromagnetic waves, hybrid ferroelectric (DFCHA)_2_AgBiI_8_ (DFCHA = 4,4-difluoropiperidinium) and others have realized low driving voltage and highly sensitive X-ray detection [87,124] (Fig. 6f). Among them, metal-free perovskite ferroelectrics have opened a new frontier for organic materials in the field of X-ray detection. All the above-reported results suggest the potential applications of hybrid perovskite ferroelectrics in energy conversion, sensors, biomedicine and storage. These findings have signified the exploration of HOIPFs toward high-performance photo-electronic and flexible devices by chemical design. Table 3 shows the captions of some abbreviations and quantities used in this article.

**Table 3.** The captions of abbreviations and quantities.

		
	Abbreviations and quantities	Caption
		
Special figures	*d*_33_	Piezoelectric coefficient
	*d*_x5_	Piezoelectric coefficient (calculated in different direction)
	*E*_c_	Coercive field
	eC	Elastocaloric
	EC	Electrocaloric
	*g*_33_	Piezoelectric voltage coefficient
	*P*_r_	Remnant polarization
	*P*_s_	Spontaneous polarization
	*s*_44_	Elastic compliance tensor
	*T*_C_	Curie temperature
	Δ*S*_EC_/Δ*E*	Isothermal electrocaloric strengths
	Δ*T*_EC_/Δ*E*	Adiabatic electrocaloric strengths
Abbreviation terms	CPL	Circularly polarized light
	EQE	External quantum efficiency
	HOIP	Hybrid organic–inorganic perovskite
	HOIPFs	Hybrid organic–inorganic perovskite ferroelectrics
	*I−V*	Current−voltage
	*J–V*	Current density−voltage
	LCP/RCP	Left-handed/right-handed circularly polarized
	*P−E*	Polarization electric field
	PFM	Piezoresponse force microscopy
	PLQY	Photoluminescence quantum yield
	PSCs	Perovskite solar cells
	PXRD	Powder X-ray diffraction
	0D	Zero-dimensional
	1D	One-dimensional
	2D	Two-dimensional
	3D	Three-dimensional
Organic components	AMP	4-(aminomethyl)piperidinium
	ATHP	4-aminotetrahydropyran
	α-PEA	α-phenylethylamine
	BA	n-butylammonium
	DABCO	*N*-*N*'-diazabicyclo(2.2.2)octonium
	DFCHA	4,4-difluorocyclohexylammonium
	DIPAB	diisopropylammonium bromide
	DMAA	*N,N*-dimethylallylammonium
	D-MNP3	Deuterated MDABCO–NH_4_(PF_6_)_3_
	FA	Formamidinium
	HMTA	Hexamethylenetetramium
	MA	Methylammonium
	MDABCO	*N*-methyl-1,4 diazoniabicyclo(2.2.2)octane
	MNP3	MDABCO–NH_4_(PF_6_)_3_
	MP	Methylphosphonium
	ODABCO	*N*-hydroxy-*N*′- diazabicyclo(2.2.2)octonium
	PMN–PT	Pb(Mg_1/3_Nb_2/3_)O_3_–PbTiO_3_
	PTMA	*N,N,N*-trimethylanilinium
	PZT	Pb(Zr,Ti)O_3_
	RM3HQ	*R*-*N*-methyl-3-hydroxylquinuclidinium
	Sm–PMN–PT	Sm-doped Pb(Mg_1/3_Nb_2/3_)O_3_–PbTiO_3_
	TMAEA	2-trimethylammonium ethyl ammonium
	TMBM	Trimethyl-bromidemethyl ammonium
		
	TMCM	Trimethylchloromethyl ammonium
	TMFM	Trimethylfluoromethyl ammonium
	[(CH_3_)_3_NOH]^+^	Trimethylammonium-*N*-oxide
	C(NH_2_)_3_^+^	Guanidium
	(CH_3_)_4_N^+^	Tetramethylammonium
	[^–^O_2_C(CH_2_)_4_CO_2_^–^]	Succinate
	[(CH_3_)_3_NCH_2_CH_2_OH]^+^	(2- hydroxyethyl)trimethylammonium
	3-AP	3-ammoniopyrrolidinium
	3-AQ	3-ammonioquinuclidinium
Multi-axial feature	Multiple polar axes	
Quasi-spherical strategy	Modification of spherical cations into quasi-spherical geometry	
Momentum matching	Motion momentum of anions and cations and the adaptation and balance of their intermolecular interaction	
Spatial confinement effect	The interaction of anions and cations in the confined environment formed by themselves	

## SUMMARY AND OUTLOOK

HOIPFs, with tunable flexible structures, environmentally friendly substances and diverse functional properties, are a great complement to conventional inorganic ferroelectrics. This review summarizes their diverse structures and functional applications in optoelectronics and related functional applications. Plentiful hybrid perovskite ferroelectrics that exhibit excellent ferroelectric properties and appropriate semiconducting provide a solid foundation for molecular ferroelectrics in energy-conversion applications. Although some encouraging results have been obtained by chemical modification in these areas, future exploration of high-performance molecular ferroelectrics and effective chemical design approaches should be of continuing importance:

- Excellent piezoelectric performance with larger *d*_33_, *P*_s_, higher *T*_C_ and more polar axes was the goal of hybrid perovskite ferroelectrics. Although some experimental results have shown great piezoelectric performance, how the organic cations and the inorganic framework affect the properties of piezoelectric has not been studied clearly. A combination of theory and experiments would be used to quantitatively predict piezoelectric properties. In the future, we can also follow several experiential rules. The plastic phase-transition, multi-axis and solid solution scheme (doped and mixed) will help us to design piezoelectric HOIPFs with higher efficiency.
- Searching for hybrid perovskite ferroelectric semiconductors with superior photoelectric performance. High-quality and large-sized crystals or thin films with semiconducting properties and sustainable stability are expected. In particular, how to optimize a high-quality film is very important for the improvement of performance, and it is also a challenge. Now, most reported works are Pb-based HOIPFs and some of them have conducted related research on photovoltaics and photodetection [125]. However, their photovoltaics effect and photodetector performance need to be further improved. With a bandgap of >2 eV, it is difficult to accommodate visible or even infrared light absorption/emission, losing great technological interest. 2D multi-layer lead–iodide perovskite ferroelectrics with a bandgap of <2 eV are rare. Therefore, on the one hand, utilizing chemical methods including ‘quasi-spherical theory’, introducing chiral cations and integrating spatial confinement should be effective methods to design 2D multi-layer perovskite ferroelectrics. On the other hand, optimizing thin-film growth methods such as solution, mechanical exfoliation, double-source co-evaporation, etc. is also one of the directions for their future research on flexible optoelectronic devices.
- Notably, inorganic perovskite ferroelectric materials have been proven to be applicable in the field of catalysis. This ability may come from the domain structure of ferroelectrics, piezoelectric effect, pyroelectric effect and semiconducting. For hybrid perovskite ferroelectrics, their moisture-sensitive nature largely hinders their research. But the organo–lead halide [(Pb_8_I_8_{H_2_O}_3_)^8+^(^−^O_2_C{CH_2_}_4_CO_2_^−^)_4_, (^−^O_2_C{CH_2_}_4_CO_2_^−^) = succinate] shows highly robust photocatalytic performance under aqueous conditions, bringing light to hybrid perovskite ferroelectrics [126]. The recent emergence of light-controlled polarization changes in organic ferroelectrics can also provide the possibility for light-controlled HOIPFs in future photocatalysis [127]. Furthermore, hybrid perovskite ferroelectrics with unique homochiral properties might bring forth chiral domains with different orientations. Hence, hybrid perovskite ferroelectrics may play an important role in the field of catalysis including enantioselective and photochemical reactions, which would extend the application range of hybrid perovskite ferroelectrics. Future development of hybrid perovskite ferroelectrics could be focused on the potential abilities to catalyse different chemical reactions.

Hybrid perovskite ferroelectrics combining organic and inorganic components may bring plentiful functional applications. Moreover, developing new lead-free and metal-free replacement materials has a powerful impetus because lead has toxicity. With more efforts and new approaches, we believe hybrid perovskite ferroelectrics will set off another revolution in the development of ferroelectric materials.

## Supplementary Material

nwac240_Supplemental_File
