# Supplementary material for: Ferroelectric hybrid organic–inorganic perovskites and their structural and functional diversity
Source: Natl Sci Rev. 2022 Nov 2;10(2):nwac240. doi: 10.1093/nsr/nwac240 (PMC9935996; doi:10.1093/nsr/nwac240)
Supplement: nwac240_Supplemental_File [file nwac240_supplemental_file.doc]

**Supplementary Information**

**Ferroelectric Hybrid Organic-inorganic Perovskites and Their Structural and Functional Diversity**

Tie Zhang^1,†^, Ke Xu^1,†^, Jie Li^1^, Lei He^1^, Da-Wei Fu^2^,*,Qiong Ye^1^,* and Ren-Gen Xiong^1^,*

^1^Jiangsu Key Laboratory for Science and Applications of Molecular Ferroelectrics, Southeast University, Nanjing 211189, China

^2^Institute for Science and Applications of Molecular Ferroelectrics, Key Laboratory of the Ministry of Education for Advanced Catalysis Materials, Zhejiang Normal University, Jinhua, 321004, China

***Corresponding authors**. E-mails: [dawei@zjnu.edu.cn](mailto:dawei@zjnu.edu.cn); [yeqiong@seu.edu.cn](mailto:yeqiong@seu.edu.cn); [xiongrg@seu.edu.cn](mailto:xiongrg@seu.edu.cn)

^†^Equally contributed to this work

**Table S1.** Summary of some perovskite ferroelectrics with their characteristics.

|  | Compounds | *d_33_* (pC N^−1^) | *P*_s_ (*μ*C cm^−2^) | *T*_c_ (K) | Transformation | Refs. |
| --- | --- | --- | --- | --- | --- | --- |
| ABX_3_ | [2-trimethylammonium ethyl ammonium] Pb_2_Cl_6_ |  | 1.0 | 412 | *Pma*2 to *Pmma* | 1 |
|  | (3-ammoniopyrrolidinium) RbBr_3_ |  | 2.3 | 440 | *Ia* to *Pmm* | 2 |
|  | [NH_2_NH_3_][M(HCOO)_3_]  (M=Mn, Zn) |  | 3.58/  3.48 | 350 | *Pna*2_1_ to *Pnma* | 3 |
|  | [NH_4_][Zn(HCOO)_3_] |  | 0.68 | 191 | *P*6_3_ to *P*6_3_22 | 4 |
|  | (H_2_hpz)[K(BF_4_)_3_] |  | 4.8 | 311 | *Pbc*2_1_ to *Pbcm* | 5 |
|  | bromocholine [Mn(dca)_3_] |  | 0.053 | 174 | *P*2_1_ to *P*2_1_/*m* | 6 |
| A_2_B^I^B^III^X_6_ | [(CH_3_)_3_NOH]_2_[KCo(CN)_6_] |  | 0.68 | 417 | *Cc* to *Fmm* | 7 |
|  | (RM_3_HQ)_2_RbLa(NO_3_)_6_ | 106 | 0.32 | 278 | *R*3 to *P*2_1_3 | 8 |
|  | (RM_3_HQ)_2_-NH_4_La(NO_3_)_6_ | 81 |  | 314 | *R*3 to *P*2_1_3 |  |
| A_n+1_B^Ⅱ^_n_X_3n+1_ | (cyclohexylaminium)_2_PbBr_4_ |  | 6.8 | 364 | *Cmc*2_1_ to *Cmca* | 9 |
|  | EA_4_Pb_3_Br_10_ |  | 3.5 | 384 | *C*2*cb* to *I*4*/mmm* | 10 |
|  | (benzylammonium)_2_PbCl_4_ |  | 13 | 438 | *Cmc*2_1_ to *Cmca* | 11 |
|  | (C_4_H_9_NH_3_)_2_PbCl_4_ |  | 2.1 | 328 | *Cmc*2_1_ to *Cmca* | 12 |
|  | (3-bromopropylammonium)_2_PbBr_4_ |  | 4.8 | 375 | *Cmc*2_1_ to *Cmca* | 13 |
|  | (4-aminotetrahydropyran)_2_PbBr_4_ | 76 | 5.6 | 503 | *Cmc*2_1_ | 14 |
|  | (4,4-difluorohexahydroazepine)_2_PbI_4_ |  | 1.1 | 454 | *P*2_1_ to *Cmc*2_1_ to *Imm*2 to *I*4̅2*m* | 15 |
|  | (perfluorobenzylammonium)_2_PbBr_4_ |  | 4.2 | 440 | *Cmc*2_1_ to *I*4*/mmm* | 16 |
|  | (4,4-DFPD)_2_PbI_4_ |  | 10 | 425 | *Aba*2 to *I*4*/mmm* | 17 |
|  | [3,3-difluorocyclobutylammonium]_2_CuCl_4_ |  | 0.29 | 380 | *Cc* to *P*42*/mmc* | 18 |
|  | [C_6_H_5_(CH_2_)_4_NH_3_]_2_[CuCl_4_] |  | 0.012 | 143 | *P*2_1_ *to P*2_1_*/c* | 19 |
| A′_2_A_n-1_B_n_X_3n+1_ | (C_6_H_5_CH_2_NH_3_)_2_CsPb_2_Br_7_ |  | 6.5 | 425 | *Cmc*2_1_ to *Cmca* | 20 |
|  | (C_4_H_9_NH_3_)_2_(NH_2_CHNH_2_)Pb_2_Br_7_ |  | 3.8 | 322 | *Cmc*2_1_ to *Cmcm* | 21 |
|  | [CH_3_(CH_2_)_3_NH_3_]_2_(CH_3_NH_3_)Pb_2_Br_7_ |  | 3.6 | 352 | *Cmc*2_1_ to *Cmca* | 22 |
|  | (C_4_H_9_NH_3_)_2_(C_2_H_5_NH_3_)_2_Pb_3_Br_10_ |  | 5 | 380 | *Cmc*2_1_ to *I*4*/mmm* | 23 |
|  | (ethylammonium)_2_(methylammonium)_2_Pb_3_Br_10_ |  | 3.7 | 375 | *Cmc*2_1_ to *I*4*/mmm* | 24 |
|  | (isoamylammonium)_2_(methylammonium)_2_Pb_3_Br_10_ |  | 5 | 303 | *Cmc*2_1_ to *I*4*/mmm* | 25 |
|  | (allyammonium)_2_(ethylammonium)_2_Pb_3_Br_10_ |  |  | 378 | *Cmc*2_1_ to *I*4*/mmm* | 26 |
|  | (IBA)_2_(EA)Pb_2_Br_7_ |  | 1 | 326 | *Cc* to *I*4*/mmm* | 27 |
|  | (IBA)_2_(EA)_2_Pb_3_Br_10_ |  | 3.5 | 370 | *Cmc*2_1_ to *I*4*/mmm* |  |
|  | (3-bromopropylaminium)_2_(formamidinium)Pb_2_Br_7_ |  | 4.2 | 348 | *Cmc*2_1_ to *Cmcm* | 28 |
|  | (IA)_2_(EA_)2_Pb_3_Br_10_ |  | 2.2 | 371 | *Cmc*2_1_ to *I*4*/mmm* | 29 |
| A′′A_n–1_B_n_I_3n+1_ | [*R*-1-(4-chlorophenyl)ethylammonium]_2_PbI_4_ |  | 13.96 | 483 | *P1 to P422* (pxrd) | 30 |
|  | [*S*-1-(4-chlorophenyl)ethylammonium]_2_PbI_4_ |  | 13.96 | 473 | *P1 to P422* (pxrd) |  |
| A_4_B^Ⅰ^B^Ⅲ^X_8_ | (chloropropylammonium)_4_AgBiBr_8_ |  | 3.2 | 305 | *Pc* to *Pbam* | 31 |
| AB^II^X_3_ | [Me_3_NCH_2_Cl]MnCl_3_ | 185 | 4.0 | 406 | *Cc* to *P*6_3_/*mmc* | 32 |
|  | [Me_3_NCH_2_Cl]CdCl_3_ | 220-240 | 6.0 | 400 | *Cc* to *P*6_3_/*mmc* |  |
|  | [4-nitrobenzylidene-1-aminopyridinium][PbBr_3_] |  | 3.45 |  | *Cc* | 33 |
|  | [Me_3_NCH_2_CH_2_OH]CdCl_3_ | 3 | 17.1 (P_r_) | 360 /393 | *Pna*2_1_ to *Pna*2_1_ to *P*6_3_/*mmc* | 34 |
|  | (Pyrrolidinium)MnBr_3_ |  | 6.2 | 219 | *Cmc*2_1_ to *Cmcm* | 35 |
|  | [FMeTP][Ni(NO_2_)_3_] |  | 3.0 | 413 | *Cc* to *P*6_3_/*mmc* | 36 |
|  | (1-azabicyclo[2.2.1]heptane)CdCl_3_ |  | 11.2 | 419 | *Pna*2_1_ to *P*2*c* | 37 |
|  | (3-Pyrrolinium) (CdCl_3_) |  | 5.1 | 316 | *Cmc*2_1_ to *Cmcm* | 38 |
|  | [Me_3_NCH_2_Br]MnBr_3_ | 112 | 3.05 | 415 | *Cc* to *P*6_3_*/mmc* | 39 |
|  | [C_6_H_5_N(CH_3_)_3_]CdCl_3_ | 4830 pm V^−1^(*d*_35_) | 3.6 (P_r_) |  | *Cc* to *Ama*2 | 40 |
|  | [(CH_3_)_4_P]CdCl_3_ |  | 0.43 | 348 | *P*6_3_ to *P*6_3_/*m* | 41 |
|  | (R)/(S)-3-(fluoropyrrolidinium)MnBr_3_ |  | 4.5 / 4.8 | 273 | *P*2_1_ to *C*222_1_ | 42 |
|  | (3-pyrrolinium)CdBr_3_ |  | 7.0 | 237.9/246.6 | *Cmc*2_1_ to  *Cmcm* | 43 |
|  | (R)/(S)-3‑(Fluoropyrrolidinium)MnCl_3_ |  | 5.0 / 5.4 | 333 | *P*2_1_ to *C*222_1_ | 44 |
|  | [(R and S)-FMQ]Ni(NO_2_)_3_ |  | 12 | 405 | *P*2_1_ | 45 |
|  | TMCM–CrCl_3_ |  | 3.6 | 397 | *Pc* to *P*6_3_*/m* | 46 |
|  | [(CH_3_)_3_NCH_2_I]PbI_3_ |  | 0.67 | 312 | *C*2 to *C*2*/m* | 47 |
|  | [(CH_3_)_3_NCH_2_F]ZnCl_3_ |  | 4.8 | 232 | *Pmn*2_1_ | 48 |
|  | (3-Pyrrolinium)MnCl_3_ |  | 6.2 | 376 | *Cmc*2_1_ to *Cmcm* | 49 |
|  | (R/S)-(+)-1 cyclohexylethylammonium) PbI_3_ |  | 1.2 | 373 | *P*2_1_ to *P*2_1_2_1_2_1_ | 50 |
|  | DMAACdCl_3_ | 41 | 1.9 | 339 | *Pna*2_1_ to *Pnma* | 51 |
|  | [C_5_H_9_NH_3_][CdCl_3_] |  | 1.7 | 300 | *Cc* to *Cmcm* | 52 |
|  | [Me_3_NCH_2_Cl]CdBr_3_ | 139 | 3.5 | 346 | *P*6_3_*mc* to *P*6_3_/*mmc* | 53 |
|  | [R-Bz-1-APy][PbI_3_] |  |  |  | *P*6_3_ | 54 |
|  | (TMFM)x(TMCM)1-x  CdCl_3_ |  |  |  |  | 55 |
|  | [C_4_H_10_N][CdCl_3_] |  | 3.6 | 240 | *Cmc*2_1_ | 56 |
| AB^III^X_5_ | (MV)BiBr_5_ |  |  |  | *P*2_1_ to *P*2_1_/*c* | 57 |
|  | [H_2_mdap][BiCl_5_] |  | 2.38 | 372 | *Pna*2_1_ to *Pnma* | 58 |
|  | (2-(ammoniomethyl) pyridinium)SbI_5_ |  | 4 | 360 | *Pb*2_1_*a* to *Pbca* | 59 |
|  | HDA-BiI_5_ |  | 6.2 | 379 | *Pna*2_1_ to *Pnam* | 60 |
|  | [1,4-butanediammonium]BiI_5_ |  | 1.35 | 365 | *P*2_1_ *to P*2_1_*/m* | 61 |
|  | (2Aprm)_2_SbCl_5_  (2Aprm)_2_SbBr_5_ |  |  |  | *P*2_1_ | 62 |
|  | (C_4_H_8_NH_2_)_2_[SbCl_5_] |  | 0.16 | 252 | *Pmn*2_1_ to *Pmnb* | 63 |
|  | (C_2_H_5_NH_3_)_2_BiCl_5_ |  | 1.4 | 190 | *Aba*2 to *Acam* | 64 |
|  | (C_2_H_5_NH_3_)_2_[BiBr_5_] |  | 0.5 × 10^−2^ (*P*_r_) | 160/120 | *Aea*2 to *Pca*2_1_ to *Aeam* | 65 |
|  | (MV)[BiI_3_Cl_2_] |  | 80 |  | *P*4*nc* | 66 |
| AB^III^_2_X_8_ | (TAMS)Bi_2_Cl_8_ |  | 0.08 |  | *Pna*2_1_ | 67 |
| A_3_B^III^_2_X_9_ | (N-methylpyrrolidinium)_3_Sb_2_Br_9_ |  | 7.6 | 322 | *R*3*c* to *Rc* | 68 |
|  | (N-methylpyrrolidinium)_3_Sb_2_Cl_9_ |  | 5.2 | 323 | *R*3*c* to *Rc* | 69 |
|  | (Pyrrolidinium)_3_[Sb_2_Cl_9_] |  | 0.004 | — | *R*3*m* to *P*3_1_ to *C*2 | 70 |
|  | FA_3_Bi_2_I_9_ |  | 0.04 | — | *P*6_3_*mc* to *Cmc*2_1_ to *P*6_3_/*mmc* | 71 |
|  | FA_3_Sb_2_I_9_ |  | 0.005 | — | *P*6_3_*mc* to *Cmc*2_1_ to *P*6_3_/*mmc* | 72 |
|  | MA_3_Bi_2_I_9_ |  | 7.94 | 143 | *P*2_1_ to *C*2/*c* to *P*6_3_/*mmc* | 73 |
| A_5_B^III^_2_X_11_ | (C_3_N_2_H_5_)_5_Sb_2_Br_11_ |  | 1.8 | 145 | *Pn* to *P*2_1_/*n* | 74 |
|  | (FA)_5_Sb_2_Br_11_ |  | 3.0 | 163 | *P*2_1_ to *P*2_1_/*n* | 75 |
| A(NH_4_)X_3_ | ODABCO-NH_4_Br_3_ |  |  | 360 | *R*3 to P432 | 76 |
|  | ODABCO-NH_4_Cl_3_ |  |  | 368 | *Pca*2_1_ to *P*3*m* |  |
|  | *R*/*S*-3AP-NH_4_Cl_3_ |  |  | 357 | *P*2_1_ to *P*432 |  |
|  | *R*/*S*-3AP-NH_4_Br_3_ |  |  | 384 | *P*2_1_ to *P*432 |  |
|  | *R*/*S*-3AQ-NH_4_Br_3_ |  |  | 493 | *P*2_1_ to *P*432 |  |
|  | MDABCO-NH_4_(PF_6_)_3_(MNP_3_) |  | 5.7 | 307 | *R*3 to *P*432 | 77 |
|  | D-MNP_3_ |  | 4.6 | 316 | *R*3 to *P*432 |  |

**References**

1. Zhang HY, Song XJ and Cheng H *et al*. A Three-Dimensional Lead Halide Perovskite-Related Ferroelectric. *J Am Chem Soc* 2020; **142**: 4604-8.

2. Pan Q, Liu ZB and Tang YY *et al*. A Three-Dimensional Molecular Perovskite Ferroelectric: (3-Ammoniopyrrolidinium)RbBr_3_. *J Am Chem Soc* 2017; **139**: 3954-7.

3. Chen S, Shang R and Hu KL *et al*. [NH_2_NH_3_][M(HCOO)_3_] (M = Mn^2+^, Zn^2+^, Co^2+^and Mg^2+^): structural phase transitions, prominent dielectric anomalies and negative thermal expansion, and magnetic ordering. *Inorg Chem Front* 2014; **1**: 83-98.

4. Xu GC, Ma XM and Zhang L *et al*. Disorder-Order Ferroelectric Transition in the Metal Formate Framework of [NH_4_][Zn(HCOO)_3_]. *J Am Chem Soc* 2010; **132**: 9588–90.

5. Chen XX, Zhang XY and Liu DX *et al*. Room-temperature ferroelectric and ferroelastic orders coexisting in a new tetrafluoroborate-based perovskite. *Chem Sci* 2021; **12**: 8713-21.

6. Wang SS, Chen XX and Huang B *et al*. Unique Freezing Dynamics of Flexible Guest Cations in the First Molecular Postperovskite Ferroelectric: (C_5_H_13_NBr)[Mn(N(CN)_2_)_3_]. *CCS Chemistry* 2019; **1**: 448-54.

7. Xu WJ, Romanyuk K and Zeng Y *et al*. Statics and dynamics of ferroelectric domains in molecular multiaxial ferroelectric (Me_3_NOH)_2_[KCo(CN)_6_]. *J Mater Chem C* 2021; **9**: 10741-8.

8. Shi C, Ma JJ and Jiang JY *et al*. Large Piezoelectric Response in Hybrid Rare-Earth Double Perovskite Relaxor Ferroelectrics. *J Am Chem Soc* 2020; **142**: 9634-9641.

9. Sun Z, Liu X and Khan T. *et al*. A Photoferroelectric Perovskite-Type Organometallic Halide with Exceptional Anisotropy of Bulk Photovoltaic Effects. *Angew Chem Int Ed* 2016; **55**: 6545-50.

10. Wang S, Liu X, Li L, Ji C, Sun Z and Wu Z et al. An Unprecedented Biaxial Trilayered Hybrid Perovskite Ferroelectric with Directionally Tunable Photovoltaic Effects. *J Am Chem Soc* 2019;**141**:7693-7.

11. You L, Liu F and Li H *et al*. In-Plane Ferroelectricity in Thin Flakes of Van der Waals Hybrid Perovskite. *Adv Mater* 2018; **30**: 1803249.

12. Ji C, Wang S and Li L *et al*. The First 2D Hybrid Perovskite Ferroelectric Showing Broadband White-Light Emission with High Color Rendering Index. *Adv Funct Mater* 2019; **29**: 1805038.

13. Ji C, Dey D and Peng Y *et al*. Ferroelectricity-Driven Self-Powered Ultraviolet Photodetection with Strong Polarization Sensitivity in a Two-Dimensional Halide Hybrid Perovskite. *Angew Chem Int Ed* 2020; **59**: 18933-18937.

14. Chen XG, Song XJ and Zhang ZX *et al*. Two-Dimensional Layered Perovskite Ferroelectric with Giant Piezoelectric Voltage Coefficient. *J Am Chem Soc* 2020; **142**: 1077-82.

15. Chen XG, Song XJ and Zhang ZX *et al*. Confinement-Driven Ferroelectricity in a Two-Dimensional Hybrid Lead Iodide Perovskite. *J Am Chem Soc* 2020; **142**: 10212-8.

16. Zhang HY, Zhang ZX and Song XJ *et al*. Two-Dimensional Hybrid Perovskite Ferroelectric Induced by Perfluorinated Substitution. *J Am Chem Soc* 2020; **142**: 20208-15.

17. Zhang HY, Song XJ and Chen XG *et al*. Observation of Vortex Domains in a Two-Dimensional Lead Iodide Perovskite Ferroelectric. *J Am Chem Soc* 2020; **142**: 4925-31.

18. Huang CR, Luo X nd Chen XG *et al*. A multiaxial lead-free two-dimensional organic-inorganic perovskite ferroelectric. *Nat Sci Rev* 2021; **8**: nwaa232.

19. Huang B, Wang BY and Du ZY *et al*. Importing spontaneous polarization into a Heisenberg ferromagnet for a potential single-phase multiferroic. *J Mater Chem C* 2016; **4**: 8704-8710.

20. YaoY, Peng Y and Li L *et al.* Exploring a Fatigue-Free Layered Hybrid Perovskite Ferroelectric for Photovoltaic Non-Volatile Memories. *Angew Chem Int Ed* 2021; **60**: 10598-602.

21. Li L, Shang X and Chen X, *et al.* Bilayered Hybrid Perovskite Ferroelectric with Giant Two-Photon Absorption. *J Am Chem Soc* 2018; **140:** 6806- 9.

22. Li L, Liu X and Han S *et al.* Two-Dimensional Hybrid Perovskite-Type Ferroelectric for Highly Polarization-Sensitive Shortwave Photodetection. *J Am Chem Soc* 2019; **141**:2623-9.

23. Ji C, Wang S L and Sun Z *et al.* 2D Hybrid Perovskite Ferroelectric Enables Highly Sensitive X-Ray Detection with Low Driving Voltage. *Adv Funct Mater* 2020; **30**: 1905529.

24. Liu X, Wang S and Xu Z *et al*. Polarization-Driven Self-Powered Photodetection in a Single-Phase Biaxial Hybrid Perovskite Ferroelectric. *Angew Chem Int Ed* 2019; **58**:14504-8.

25. Li M, Xu Y, and Liu Y *et al*. Giant and Broadband Multiphoton Absorption Nonlinearities of a 2D Organometallic Perovskite Ferroelectric. *Adv Mater* 2020; **32**:2002972.

26. Peng Y, Liu X and Wu Z *et al*. Exploiting the Bulk Photovoltaic Effect in a 2D Trilayered Hybrid Ferroelectric for Highly Sensitive Polarized Light Detection. *Angew Chem Int Ed* 2020; **59**:3933-7.

27. Peng Y, Bie J and Fa W *et al*. Acquiring High-TC Layered Metal Halide Ferroelectrics via Cage-Confined Ethylamine Rotators. *Angew Chem Int Ed* 2021; **60**:2839-43.

28. Wu Z, Zhang W and Li L *et al*. Bromine-Substitution-Induced High-Tc Two-Dimensional Bilayered Perovskite Photoferroelectric. *J Am Chem Soc* 2021; **143**:7593-8.

29. Ma Y, Wang J and Guo W *et al*. The First Improper Ferroelectric of 2D Multilayered Hybrid Perovskite Enabling Strong Tunable Polarization-Directed Second Harmonic Generation Effect. *Adv Funct Mater* 2021; **31**: 2103012.

30. Yang CK, Chen WN and Ding YT *et al*. The first 2D homochiral lead iodide perovskite ferroelectrics: [*R*- and *S*-1-(4-chlorophenyl)ethylammonium]_2_PbI_4_. *Adv Mater* 2019; **31**:1808088.

31. Guo W, Liu X and Han S *et al*. Room-Temperature Ferroelectric Material Composed of a Two-Dimensional Metal Halide Double Perovskite for X-ray Detection. *Angew Chem Int Ed* 2020; **59**: 13879-84.

32. You YM, Liao WQ and Zhao D *et al.* An organic-inorganic perovskite ferroelectric with large piezoelectric response. *Science* 2017; **357**: 306-9.

33. Zhao SP, Guo Y and Wang J *et al.* An inorganic-organic hybrid compound with face-sharing bromoplumbate chain: Synthesis, crystal structure, Hirshfeld surface analysis, ferroelectric and dielectric properties. *Polyhedron* 2020; **178**: 114345.

34. Deswal S, Singh S. K and Pandey R *et al.* Neutral 1D Perovskite-Type ABX_3_ Ferroelectrics with High Mechanical Energy Harvesting Performance. *Chem Mater* 2020; **32**: 8333-41.

35. Zhang Y, Liao WQ and Fu DW *et al.* The First Organic–Inorganic Hybrid Luminescent Multiferroic: (Pyrrolidinium)MnBr_3_. *Adv Mater* 2015; **27**: 3942-6.

36. Xiong YA, Sha TT and Pan Q *et al.* A Nickel(II) Nitrite Based Molecular Perovskite Ferroelectric. *Angew Chem Int Ed* 2019; **58**: 8857-61.

37. Tang YY, Xie Y and Zeng YL *et al.* Record Enhancement of Phase Transition Temperature Realized by H/F Substitution. *Adv Mater* 2020; **32**: 2003530.

38. Ye HY, Zhang Y and Fu DW *et al.* An Above-Room-Temperature Ferroelectric Organo–Metal Halide Perovskite: (3-Pyrrolinium)(CdCl_3_). *Angew Chem Int Ed* 2014; **53**: 11242-7.

39. Liao WQ, Tang YY and Li PF *et al.* Large Piezoelectric Effect in a Lead-Free Molecular Ferroelectric Thin Film. *J Am Chem Soc* 2017; **139**: 18071-7.

40. Hu Y, You L and Xu B *et al.* Ferroelastic-switching-driven large shear strain and piezoelectricity in a hybrid ferroelectric. *Nat Mater* 2021; **20**: 612-7.

41. Zhou L, Shi PP and Liu XM *et al.* An above-room-temperature phosphonium-based molecular ferroelectric perovskite, [(CH_3_)_4_P]CdCl_3_, with Sb^3+^-doped luminescence. *NPG Asia Mater* 2019; **11**: 15.

42. Gao JX, Zhang WY and Wu ZG *et al.* Enantiomorphic Perovskite Ferroelectrics with Circularly Polarized Luminescence. *J Am Chem Soc* 2020; **142**: 4756-61.

43. Li PF, Liao WQ and Tang YY et al. Unprecedented Ferroelectric–Antiferroelectric–Paraelectric Phase Transitions Discovered in an Organic–Inorganic Hybrid Perovskite. *J Am Chem Soc* 2017; **139**: 8752-7.

44. Ai Y, Chen XG and Shi PP *et al.* Fluorine Substitution Induced High Tc of Enantiomeric Perovskite Ferroelectrics: (R)- and (S)-3-(Fluoropyrrolidinium)MnCl_3_. *J Am Chem Soc* 2019; **141**: 4474-9.

45. Deng BB, Xu CC and Cheng TT *et al.* Homochiral Nickel Nitrite ABX_3_ (X = NO_2_^–^) Perovskite Ferroelectrics. *J Am Chem Soc* 2020; **142**: 6946-50.

46. Ai Y, Sun R and Zeng YL *et al.* Coexistence of magnetic and electric orderings in a divalent Cr2+-based multiaxial molecular ferroelectric. *Chem Sci* 2021; **12**: 9742-7.

47. Hua XN, Liao WQ and Tang YY *et al.* A Room-Temperature Hybrid Lead Iodide Perovskite Ferroelectric. *J Am Chem Soc* 2018; **140**: 12296-302.

48. Chen L, Liao WQ and Ai Y *et al.* Precise Molecular Design Toward Organic–Inorganic Zinc Chloride ABX_3_ Ferroelectrics. *J Am Chem Soc* 2020; **142**: 6236-43.

49. Ye HY, Zhou Q and Niu X *et al.* High-Temperature Ferroelectricity and Photoluminescence in a Hybrid Organic–Inorganic Compound: (3-Pyrrolinium)MnCl_3_. *J Am Chem Soc* 2015; **137**: 13148-54.

50. Hu Y, Florio F and Chen Z *et al.* A chiral switchable photovoltaic ferroelectric 1D perovskite. *Sci Adv* 2020; **6**: eaay4213.

51. Wang ZX, Zhang H and Wang F *et al.* Superior Transverse Piezoelectricity in a Halide Perovskite Molecular Ferroelectric Thin Film. *J Am Chem Soc* 2020; **142**: 12857-64.

52. Zhang Y, Ye HY and Zhang W *et al.* Room-temperature ABX_3_-typed molecular ferroelectric: [C_5_H_9_–NH_3_][CdCl_3_]. *Inorg Chem Front* 2014; **1**: 118-23.

53. Liao WQ, Tang YY and Li PF *et al.* Competitive Halogen Bond in the Molecular Ferroelectric with Large Piezoelectric Response. *J Am Chem Soc* 2018; **140**: 3975-80.

54. Duan HB, Zhao HR and Ren XM *et al.* Inorganic–organic hybrid compounds based on face-sharing octahedral [PbI_3_]∞ chains: self-assemblies, crystal structures, and ferroelectric, photoluminescence properties. *Dalton Trans* 2011; **40**: 1672-83.

55. Liao WQ, Zhao D and Tang YY *et al.* A molecular perovskite solid solution with piezoelectricity stronger than lead zirconate titanate. *Science* 2019; **363**: 1206-10.

56. Xu WJ, He CT and Ji CM *et al.* Molecular Dynamics of Flexible Polar Cations in a Variable Confined Space: Toward Exceptional Two-Step Nonlinear Optical Switches. *Adv Mater* 2016; **28**: 5886-90.

57. Bi W, Leblanc N and Mercier N *et al.* Thermally Induced Bi(III) Lone Pair Stereoactivity: Ferroelectric Phase Transition and Semiconducting Properties of (MV)BiBr_5_ (MV= methylviologen). *Chem Mater* 2009; **21**: 4099-101.

58. Wang Y, Shi C and Han XB. Organic–inorganic hybrid [H_2_mdap][BiCl_5_] showing an above-room-temperature ferroelectric transition with combined order–disorder and displacive origins. *Polyhedron* 2017; **133**: 132-6.

59. Li PF, Tang YY and Liao WQ *et al.* A semiconducting molecular ferroelectric with a bandgap much lower than that of BiFeO_3_. *NPG Asia Mater* 2017; **9**: e342.

60. Zhang HY, Wei Z and Li PF *et al.* The Narrowest Band Gap Ever Observed in Molecular Ferroelectrics: Hexane-1,6-diammonium Pentaiodobismuth(III). *Angew Chem Int Ed* 2018; **57**: 526-30.

61. Liu YH, Peng H and Liao WQ. A lead-free bismuth iodide organic–inorganic ferroelectric semiconductor. *Chem Commun* 2021; **57**: 647-50.

62. Owczarek M, Szklarz P and Jakubas R. Towards ferroelectricity-inducing chains of halogenoantimonates(iii) and halogenobismuthates(iii). *RSC Adv* 2021; **11**: 17574-86.

63. Ksiądzyna M, Gągor A and Piecha-Bisiorek A *et al.* Exploring a hybrid ferroelectric with a 1-D perovskite-like structure: bis(pyrrolidinium) pentachloroantimonate(iii). *J Mater Chem C* 2019; **7**: 10360-70.

64. Piecha-Bisiorek A, Gągor A and Jakubas R *et al.* Ferroelectricity in bis(ethylammonium) pentachlorobismuthate(iii): synthesis, structure, polar and spectroscopic properties. *Inorg Chem Front* 2017; **4**: 1281-6.

65. Jakubas R, Ga̧gor A and Winiarski M. J *et al.* Ferroelectricity in Ethylammonium Bismuth-Based Organic–Inorganic Hybrid: (C_2_H_5_NH_3_)_2_[BiBr_5_]. *Inorg Chem* 2020; **59**: 3417-3427.

66. Leblanc N, Mercier N and Zorina L *et al.* Large Spontaneous Polarization and Clear Hysteresis Loop of a Room-Temperature Hybrid Ferroelectric Based on Mixed-Halide [BiI_3_Cl_2_] Polar Chains and Methylviologen Dication. *J Am Chem Soc* 2011; **133**: 14924-7.

67. Xu G, Li Y and Zhou WW *et al.* A ferroelectric inorganic–organic hybrid based on NLO-phore stilbazolium. *J Mater Chem* 2009; **19**: 2179-83.

68. Sun Z, Zeb A and Liu S *et al*. Exploring a Lead-free Semiconducting Hybrid Ferroelectric with a Zero-Dimensional Perovskite-like Structure. *Angew Chem Int Ed* 2016; **55**: 11854-8.

69. Ji C, Sun Z and Zeb A *et al*. Bandgap Narrowing of Lead-Free Perovskite-Type Hybrids for Visible-Light-Absorbing Ferroelectric Semiconductors. *J Phys Chem Lett* 2017; **8**: 2012-8.

70. Wojciechowska, M, Gągor A and Piecha-Bisiorek A *et al*. Ferroelectricity and Ferroelasticity in Organic Inorganic Hybrid (Pyrrolidinium)_3_[Sb_2_Cl_9_]. *Chem Mater* 2018; **30**: 4597-608.

71. Szklarz P, Gągor A and Jakubas R *et al*. Lead-free hybrid ferroelectric material based on formamidine: [NH_2_CHNH_2_]_3_Bi_2_I_9_. *J Mater Chem C* 2019; **7**: 3003-14.

72. Szklarz P, Jakubas R and Gągor A *et al*. [NH_2_CHNH_2_]_3_Sb_2_I_9_: a lead-free and low-toxicity organic–inorganic hybrid ferroelectric based on antimony(iii) as a potential semiconducting absorber. *Inorg Chem Front* 2020; **7**: 1780-9.

73. Kamminga ME, Stroppa A and Picozzi S *et al*. Polar Nature of (CH_3_NH_3_)_3_Bi_2_I_9_ Perovskite-Like Hybrids. *Inorg Chem* 2017; **56**: 33-41.

74. Piecha A, Pietraszko A and Bator G *et al*. Structural characterization and ferroelectric ordering in (C_3_N_2_H_5_)_5_Sb_2_Br_11_. *J Solid State Chem* 2008; **181**: 1155-66.

75. Mencel K, Gągor A and Jakubas R *et al*. Ferroelectricity in a lead free organic–inorganic 0D hybrid: formamidinium bromoantimonate(iii). *J Mater Chem C* 2020; **8**: 5025-8.

76. Ye HY, Tang YY and Li PF *et al*. Metal-free three-dimensional perovskite ferroelectrics. *Science* 2018; **361**: 151-5.

77. Choi H S, Li S and Park I H *et al*. Tailoring the coercive field in ferroelectric metal-free perovskites by hydrogen bonding. *Nat Commun* 2022; **13**: 794.
